# The Lawyer and the Doctor*Address to the Central Texas Medical Association, at Waco, Texas, July 12th, 1899.

**Published:** 1899-08

**Authors:** Jno. G. Winter

**Affiliations:** Waco, Texas


### TEXAS MEDICAL JOURNAL.

ESTABLISHED JULY, 1885.

PUBLISHED MONTHLY.—SUBSCRIPTION $1.00 A YEAR.

VOL. XV. AUSTIN, AUGUST, 1899. No. 2.

### Original Contributions.

For the Texas Medical Journal.

### The Lawyer and the Doctor.*

BY HON. JNO. G. WINTER, WACO, TEXAS.

*Mr. President and Gentlemen of the Association:*

I will endeavor, as best I can, to respond to the kind and flattering invitation to present "a paper" on the subject assigned, which formulated with that perspicuity characteristic of your prescriptions, is entitled "Medicus et chirurgus sapiens et jurisconsaltus et patronis;" and so contribute my mite to the evening's entertainment. Whatever of hesitancy or responsibility I might otherwise feel in treating a subject with so formidable a front, is minimized by the reflection that my paper is neither intended or expected to be exhaustive, nor more than a mark for the learned and eloquent gentlemen who are programmed to discuss the text. When we reflect that the disciples of the two professions,—as all the world knows, conspicuously live and have their being in the sombre atmosphere of deep meditation and profound thought, the gravity of the subject is apparent.

The *Augurs,* who for more than half a thousand years so largely swayed the councils of Greece and of Rome, in the halcyon days of

*Address to the Central Texas Medical Association, at Waco, Texas, July 12th, 1899.

those mighty and cultured peoples smiled when they met. Wherefore, is conjectural. It has been suggested, and by the moderns widely accepted, that it was for the reason that each may have had the self-consciousness that possibly, neither jointly or severally, were they quite so wise as they seemed or as their fellow citizens supposed them to be. It has also been advanced, and that school, too, has its followers, that the alleged habit is reasonably explicable upon the theory that the "smile," so often an accompaniment of latter day greeting, had, even in that early stage of civilization the same technical significance as now. But these things are enveloped in the shadows of the centuries, and we can but vaguely speculate. Be as it may—howsoever insoluble the problem,—I nevertheless suggest for at least a touch of discussion, is there anything in the text, "The doctor *and* the lawyer" suggestive of the Roman Augur or his brother bore of Greece?

The doctor and the lawyer meet and greet each other, and perhaps smile—but none gainsay but that it is the smile of unsophisticated innocence. It is not permissible that I speak of your profession with the freedom and fulness that I may discuss my own, neither would it become me to laud my own—yet I may ask, Does the lawyer doubt or question?

With serene confidence he will swallow the mystical mixtures of his medical brother. Solemnly prescribed, "Sodium chloride one drachm; tincture cacus q. s.; aqua pura two double drachms," will restore the vigor of his weary brain and o'ertaxed nerves.

"Faith is a higher faculty than reason."
The lawyer is trustful for that he is guileless.
To the truly good—all is good and true.

Blackstone says: "For the gentlemen of the faculty of physic, I must frankly own that I see no special reason why they in particular should apply themselves to the study of the law, unless indeed, in common with other gentlemen, and to complete the character of general and extensive knowledge—a character which their profession above others, has remarkably deserved." They will give me leave, however, to suggest that such study might very frequently be of use to families under medical care, if the physician were acquainted with the doctrine of *last wills and testaments.*

Medicine and the law are jealous mistresses. Both require, theoretically, of their votaries, the fullest preparatory learning and culture, but once the livery is donned, neither will tolerate a rival. Blackstone was a poet, but was compelled, perforce, to abandon the flowery paths. His last lines indicate the cause of his desertion. While we must admire the sonorous rythm, we can but hope for his reputation as a bard, that his notes differ from those of the swan in this that his last are not his sweetest. He tell us,

"Knowledge in law care only can attain,
Where honor is purchased at the price of pain;
If loitering, up the ascent we cease to climb,
No straits of labor can redeem the time;
Industrious study only wins by slow degrees,
True sons of Coke can ne'er be sons of ease."

The muse does not confine her favors to the "wig and gown," and the doctor, with no unskillful touch sometimes mixes poetry with his pills.

Amidst amputated legs and arms, crooked spines and "lovely tumors," one of your number, here present, has said,

"There is music in all of nature's songs,
If for music you do but care;
There is a concert, free for every one,
If he will only but stop and hear."

"And lo! the winged messenger fetched an 'Olive' branch."

If assiduous and unremitting study and application is necessary to proficiency in the law, how much more so, how infinitely more so, gentlemen, are they essential in *your* profession. God has given us this complex frame as our mortal dwelling house; you, by your own volition have assumed the fearful responsibility of its repair and preservation.

You have all, no doubt, observed the manner in which lawyers are accustomed to dispose their time. Their rules in this regard are of ancient origin—back to a period whereof the memory of man runneth not to the contrary. Lord Coke more recently has formulated them:

"Six hours in sleep;
In law's grave study, six;
Four spend in prayer,
The rest on nature fix."

The division is nominally, but really not different, with the medical fraternity. Lawyers are told of doctors that *they* give, or are supposed to give,

"Seven hours to study,—
To soothing slumber seven;
Ten to the world—all to Heaven."

But whatever of the *hours* or howsoever closely observed, we learn to know in time—both the doctor and the lawyer—how little, at the best, we do acquire.

In the programe of your proceedings it is noted that one of your most eminent and accomplished fraters, one whose reputation is not limited to State lines, would tell you what he did *not* know of a certain branch of medical science. Notwithstanding his great learning, observation and experience, I could but reflect, that if he purposed to furnish "a bill of particulars," that your adjournment would be indefinitely postponed.

Professor Simon Newcomb says: "It may be wondered how—after devoting so much work to the study of the heavens, anything can remain for astronomers to find out. It is a curious fact that altho' they were never learning so fast as at the present day, yet there seems to be more to learn *now* than there *ever* was before." So likewise, it is with your profession and ours. Alas! our knowledge is finite—what we do not know is infinite.

### THE DOCTOR AND THE LAWYER.

Medical jurisprudence, in the more restricted and common use and acceptation of the term, is that science which applies the principles and practice of medicine and surgery to the elucidation of doubtful questions in courts of justice, and treats of matters requiring medical knowledge and skill, as well as knowledge of law. It is a science which involves the union of the two great secular professions, and creates a bond between the doctor and the lawyer. Their learning here converges to a common point, towit:

"The elucidation of doubtful questions in courts of justice."

In ordinary, daily practice, there is no conflict—no attrition.

Each may win and wear the laurels of victory and success, and yet pluck no leaf from the other's wreath. Both wear the cypress of defeat, and yet may unselfishly condole together—there is no disputed territory, and they are free unjealously to appreciate and enjoy each other. Their relations are untrammeled, and their lines are parallel to the end, for, as has been wittily said, "Doctors bury their mistakes beneath the sod—lawyers hang theirs in the air."

Anomalous it is, that it is only when the disciples of Hippocrates and Galen, and those of Coke and Blackstone, unite in a common purpose, towit, to "elucidate doubtful questions," and to aid the blind goddess to light, that the battle royal is on betwixt them, and sometimes indeed it is a battle royal.

We have all participated in, or at least observed such "elucidations."

It is when the medical expert is on the witness stand, an expectant jury before him, with skillful lawyers, specially read and "crammed" for the occasion, as interlocutors, that the "elucidation" proceeds and the effulgent rays of scientific truth wrap about and envelope the facts and the jury, until the one is fused into an unrecognizable conglomeration, and the other, dazed and befuggled with hypothetical cases—bearing scarcely family likeness to the real one,—retire to consider their verdict, in the light of a luminous charge by the court, which is forbidden to be either abstract or concrete. As illustrative, it is tradition—and said to be true—that in a famous and hotly contested poisoning case, in which the accomplished Dr. Ashbel Smith, ambassador of Texas to the court of St. James, was the chief medical expert,—the bewildered and exhausted jury returned the astounding verdict: "The deceased came to his death by hypothesis."

Thus the doctor and the lawyer conjunctively *"elucidate."*

The situation of such a witness is ofttimes a trying one for a conscientious man. He recognizes that he is called upon to give only his wrought opinions. He cannot shield himself behind those authorities which have guided him in his studies, nor can he rely upon the teachings of his oral lectures; to do so would be to give the opinions of absent witnesses beyond the reach of cross-examination,—he can give only his *own* opinions, deduced from all such sources of information and from his own experience.

It perhaps seldom occurs that the medical expert takes the witness stand with entirely unbiased mind. He is not in court of his own volition. He is there in response to a subpœna from the party to the suit whose interest it is to have him there, and it is rarely that he is called to attend court, unless in some manner his, at least, prima facie opinions concerning the case have been previously ascertained.

I imagine that in the great majority of instances where experts are called there is room for honest differences of opinion as to some one or more phases of the case. The lawyers engaged to assist in "elucidating," usually smartly, if superficially, inform themselves as to the particular questions concerning which the doctor is expected to testify, and unless the witness preserves both his wit and his temper the "elucidation" pro and con, not unseldom raises so much of learned mists that the truth is fatally obscured. I have had no inconsiderable experience in suits involving testimony of this character, and my observation teaches me that no better course can be adopted by such witnesses, both in direct and under fire of cross-examination, than that advised by an eminent writer of your profession, who says: "In all expert evidence the witness must never for a moment transgress the limits of scientific accuracy, nor with the warmth of a partisan or the desire to do the best that he can for his case, permit himself to color or distort, directly or indirectly, the hard, straight and inflexible lines of well attested scientific facts, such as he would never dream of disputing, coloring or distorting before a learned society."

If a witness, in heat of the moment, allows himself to break away from the safe course embraced in this advice, the lawyer will be pretty sure to catch him away from his base. I have heard doctors say that lawyers are sometimes "right mean" about that.

How far courts can compel the attendance of medical experts, and what are the privileges of such witnesses, are questions concerning which the courts have differed in different jurisdictions, but there are certain lines upon which all substantially agree. In the first place there is broad distinction between the obligations of the expert in criminal and in civil cases. In the first, he owes a duty to the State, either for the conviction of the guilty or the vindication of the innocent. It is a public duty that he must discharge. Yet even in those cases he cannot be required, without "extra compensation," as distinguished from the usual witness fees prescribed by law, to do more than testify concerning facts that have come to his knowledge and to give his opinions based upon those facts. He cannot be required to inform himself as to facts, any more than can any other witness. It has been well said in a case before the Supreme Court of Indiana (8 Ind., 467):

"It is as much the duty and interest of every citizen to aid in prosecuting crime, as it is to aid in subduing any domestic or foreign enemy, and it is equally the interest and duty of every citizen to aid in furnishing to all, high and low, rich and poor, every facility for a fair and impartial trial when accused, *for none are exempt from the liability to accusation and trial.*"

In the absence of controlling statutes, experts, whether medical or in other sciences, should be required, in *criminal* cases, without extra compensation, to testify to matters involving professional or special knowledge and skill. His protection against unreasonable demands lies in the fact that he cannot be required to make any personal examination or preliminary preparation, or indeed to attend throughout a trial for the purpose of listening to the testimony. His opinion can be had on a hypothetical case. But as a general rule, something more than that is desired, and that something cannot be demanded without suitable compensation.

The question is not an open one in Texas.

To illustrate, it has been held by our highest courts that a physician who had made a post-mortem examination, and thus acquired knowledge of the facts, could be required to testify. It is said "the court can compel a witness to testify to the result of a post-mortem examination, and it is to be regretted that a member of a profession so distinguished for liberal culture and high sense of honor and duty should refuse to testify in a cause pending before the courts of his country, involving the life and liberty of a fellow being and the rightful administration of the laws of a common country. * * * A medical expert could not be compelled to *make* a post-mortem examination unless previously paid for it, but an examination having already *been* made by him, he could be compelled to disclose the result of that examination." It does not appear but that these principles would apply with equal force to any case involving abnormal conditions that might bear upon the question of criminal responsibility.

The courts are not more exacting in this respect with the medical than with the legal profession. The judge may assign the ablest of the bar to defend a prisoner charged with felony and who is impecunious, and I have never known a lawyer wilfully to decline the task or fail to give his best efforts under such circumstances.

Indeed the lawyer's obligation requires that he *give* his service for the vindication of the right, even in civil cases, when, through poverty, the person seeking his aid is unable to compensate him. A not inapt illustration of the fidelity of the lawyer to his client in these naked trusts, is afforded in the laudable persistence of H. P. Jourdan, Esq., a young member of the Waco bar, in the case of the State vs. Ford, now attracting no little interest in this community.

My observation, however, is that in this unrequited field the doctor outranks the lawyer as dollars do dimes in our bank accounts.

Compensation of experts in criminal cases is not applicable to civil causes, where private interests alone are involved. In actions of this class, however, the doctor is not exempted from the duty, common to other citizens, of testifying to facts within his knowledge, but he cannot be required to give his opinion on a matter concerning which he is peculiarly conversant. His knowledge is his property and he is entitled to compensation for its use. Upwards of three hundred years ago the British parliament enacted a statute providing that a witness of the character under discussion should "have tendered to him, according to his countenance, his reasonable charges." Perhaps that statute has evolved the wise faces which are characteristic of the faculty.

While the question of amount paid or agreed to be paid cannot in the least affect the course of judicial proceedings, yet it does, in some degree, and perhaps unavoidably, affect the credit of the witness with the jury. Without adequate compensation, as a rule, the testimony of competent experts could not be secured, and yet the suspicion in the minds of the average juror that the witness is too biased to be fully credited would not infrequently tend to defeat a proper verdict. The question is difficult of satisfactory solution. Possibly a step in the right direction would be the enactment of a law requiring experts to be appointed under direction of the court, the compensation to be fixed and secured, to be paid by the losing party.

The lawyer—when he is in health—has at least one advantage over the doctor. He is exempt from the somewhat unpleasant and yet quite possible dilemma, of having at some time to determine whether he will honorably become the guest of the county, or, with at least, a restless conscience, continue to dine at home.

There have been, and will be cases, when the physician placed upon the witness stand must ask himself, "How far am I at liberty to answer?"

The lawyer—by the law of adjudicated cases, is shielded from disclosing professional confidential communications. He cannot be compelled to divulge the secrets of his client; indeed, I doubt if he would be allowed to do so. But it is not so with the physician, or the minister of the gospel; even the confessional is without legal safeguard.

No law in Texas protects the physician in this regard, and yet the confidences reposed in him are frequently of the most sacred character.

It is wrong that the physician should be even remotely exposed to penalties for refusing to answer from the witness stand concerning facts in any manner coming to his knowledge in the unrestrained intercourse incident to the sick room. The family physician fills an office sacred in its character and the law should shield him from the pryings of the court room—he should not be left to his unsupported sense of honor and propriety. It is in the extreme, unjust to him that he be exposed to the possible alternative of betraying the trusts in his keeping or else be held for contempt of court, possibly committed to prison. "Trifles light as air" may be made the common jest of a thoughtless public, and by exaggeration or perversion, embitter lives.

The true physician realizes this. Twenty-five centuries have elapsed since Hippocrates lived. The oath ascribed to that "Father of medicine," provides, among other things, "Whatever in connection with my professional practice, or not in connection with it, I see or hear in the life of men which ought not to be spoken of abroad I will not divulge, as reckoning that all such should be kept secret. While I continue to keep this oath unviolated, may it be granted to me to enjoy life and the practice of the art, respected by all men in all times. But should I trespass, and violate this oath, may the reverse be my lot."

All eminent writers of your profession in discussing the rulings of the courts which hold that medical men enjoy no special privileges with regard to secrets of a professional character say: "This is the law; and however it may be defended on legal grounds, we hope that there are not a few medical men who would prefer to sacrifice their personal liberty to their honor. It seems a monstrous thing to require that secrets affecting the honor of families, and perhaps confided to the medical adviser in a moment of weakness, should be dragged into the garish light of a law court, there to be discussed and made joke of by rude tongues and unsympathetic hearts."

Many States of the Union have recognized the propriety of guarding the physician as well as the lawyer in the matters to which I have referred, and have enacted laws to that end. Among such States are Arkansas, California, Colorado, Dakota, Indiana, Iowa, Kansas, Michigan, Minnesota, Missouri, Montana, Nevada, New York, Ohio, Oregon, Utah, Washington, Wisconsin, Wyoming, and perhaps others.

In the early days of the Twenty-fifth Texas Legislature, I prepared a bill upon this subject, at the request of a medical friend. It was introduced, but seems to have died in committee.

The bill was an amendment to Article 3785, of the Revised Statutes and provided: "No person duly authorized to practice physic or surgery, shall be allowed to disclose any information which he may have acquired in attending any patients in a professional character, unless such information be obtained for the preannounced purpose of elucidating some question of fact, in a court of justice."

That the physician should thus be shielded is, to my mind, too clear for disputation.

In glancing over your published programme, I have mentally likened your gatherings to the councils of defenders of a beleagured city, considering the latest reports regarding the movements of the threatening foe, and devising how best to meet his advances. To you is entrusted the task of defending the citadel wherein man dwells, and of meeting the dread attacks of time and disease. You come, with your several and varied experiences, from that everlasting conflict between life and death, and here you discuss how best to hinder and delay that enemy whose final victory can at best be but postponed. The objective point of all your labors is the alleviation of human suffering, the preservation and prolongation of human life. While you are struggling with those vital problems, forging here some new weapon of attack, welding there some new armor of defense, the world moves in its busy current of affairs, relying upon your vigilance to guard the outposts; dependent upon your skill to oust the enemy should he enter. A sense of these responsibilities can but be ennobling; and must stretch to the highest tension every faculty of mind to keep abreast the onward march and development of your science. We are taught to know that in a sound body is alone to be found the sound and balanced mind, and it follows that the life of the Republic is largely in your keeping, for it is in the union of physical and mental vigor in people that great States are founded and maintained, that civilization is broadened and developed.

Even the lawyers, who have ever been the jealous guardians of civil liberty, depend upon the doctors for that health which is essential to their full usefulness.

> "A wise physician, skilled our wounds to heal
> Is more than armies to the public weal."

But I trespass. Mr. President, truly, indeed, is yours an exalted calling. What room can there be in the soul of the true physician for other than the highest, the noblest and the loftiest sentiments.

As the most malignant germs wither and perish in the atmospheric purity of altitudes, so, it impresses me, the baser instincts of weak humanity must be eliminated from the heart of him whose life's work it is to minister to the suffering; who is so drawn to contemplate the frailty and the loftiness of man; the eternal mystery of life and death; the spirit's flight from its mortal tenement.

DISCUSSION.

Dr. D. R. Wallace said: I endorse the paper. I have had some experience along the lines discussed in this paper. I never give an intimation to any litigant as to what my testimony will be until I have heard the evidence under oath.

The importance of that determination was exemplified in the Burt case. One witness gave testimony under oath altogether different from that stated to me previously. There are more insane men hung than escape unjustly by the insanity dodge.

Dr. Howard said: I think the law of secrets of a professional character having to be divulged should be revised. I think great care should be exercised to protect the innocent insane criminal.

Dr. Ghent said: A doctor should not become a partisan in a case. Truth should be his polar star. An attorney cannot trip a man who confines himself to the truth. Don't become an advocate. The witness is often in an unpleasant situation. I think secrets given a doctor by a patient should be held sacred in his breast and no law should force him to tell it. A case in my town, of grave offense; plea insanity. The man was given belladonna to confuse his eye sight, etc. Sent into court to fool the doctors and did fool three or four; I thought, and swore he was a sane man. He said going back to jail convicted that this was the hardest crowd to fool he ever saw. He is now serving a term in the state prison. I do not think because a man killed another in the Christian town of Dallas last night that the atrocity of the crime and publicity indicate that the murderer was insane. I do not think that the murderers of the Humphries were insane. I think they and other criminals of like character should be hung as high as Haman.

Dr. Wallace: Dr. G. misunderstood me. An alienist does not form an opinion of insanity or sanity by one fact or one circumstance.

Dr. Ghent: Suppose a lady should give a doctor information that would implicate her; could the court force him to tell the secret?

Col. Winter: So many circumstances may surround that as to make me unable to answer it yes or no.

Dr. Wallace: The courts generally throw ample protection around the physician and his patient.

Mr. Geo. Graves: I had a question presented to me by doctors in reference to two prominent people in a divorce case. The question was, whether or not the doctor should be forced to go to court and testify as to what he had learned in his capacity as a physician. They were advised that the doctor must go and testify. A law permitting a doctor not to tell of any secret obtained as a doctor should have some modification. Doctor's testimony might sometimes bear very materially as to the guilt or innocence of the accused. A law of that kind should be modified to a certain extent.

